# Enhanced diversity of human immune cells in a PBMC-engrafted NSG-SGM3-IL15-DKO (S15-DKO) immunodeficient mouse model

**DOI:** 10.3389/fimmu.2026.1967450

**Published:** 2026-10-27

**Authors:** Xiaoqing Zheng, Michael A. Brehm, Leandro Salati D’Abronzo, Guoxiang Yang, Jessica K. Wong, Beau Parry, Jiwon Yang, James G. Keck, Li-Chin Yao

**Affiliations:** 1 Department of Innovation & Product Development, The Jackson Laboratory, Sacramento, CA, United States; 2 Diabetes Center of Excellence, University of Massachusetts Chan Medical School, Worcester, MA, United States

**Keywords:** bispecific T cell engagers, humanized mouse model, immunotherapy, myeloid cells, natural killer cells, NSG-SGM3-IL15-DKO (S15-DKO), peripheral blood mononuclear cell (PBMC)

## Abstract

**Background:**

Peripheral blood mononuclear cell (PBMC)-humanized mouse models are critical tools for preclinical evaluation of cancer immunotherapies, yet current models offer limited recapitulation of human immune diversity, particularly myeloid and natural killer (NK) cells. To address this limitation, we generated a novel immunocompromised mouse strain NSG-SGM3-IL15-DKO (S15-DKO) combining the NSG-MHC I/II double-knockout (DKO), which delays xenogeneic graft-versus-host disease, with the NSG-SGM3-IL15 human cytokine-expressing strain. Here, we evaluated PBMC engraftment and established a humanized immune-oncology model to test bispecific T cell engager (BiTE) efficacy and cytokine release in S15-DKO mice compared to DKO mice.

**Methods:**

S15-DKO and DKO mice were engrafted with 1x107 human PBMCs from various healthy donors, with or without prior exposure to Raji lymphoma cells or irradiation. Retro-orbital (RO) blood (and spleen) was collected to assess human immune cells engraftment by flow cytometry. Irradiated, Raji-bearing S15-DKO and DKO mice were treated with PBS or anti-CD3xCD19 BiTE. Bioluminescence imaging was performed to measure tumor burden, and plasma was collected to measure cytokine levels.

**Results:**

S15-DKO mice engrafted with PBMCs from multiple donors supported significantly higher levels of human CD45^+^ cells, CD4^+^ and CD8^+^ T cells, and NK cells compared to DKO mice without the need for irradiation, with body weight changes and survival comparable to DKO controls. B cells, dendritic cells, and macrophages also showed significantly improved engraftment in S15-DKO mice compared to DKO mice with certain PBMC donors. Engraftment of PBMCs in S15-DKO mice bearing Raji lymphoma cells was also higher compared to DKO mice, with significantly elevated numbers of NK cells and dendritic cells in addition to T cells. Preconditioning the S15-DKO mice with irradiation prior to PBMC engraftment further enhanced immune cell reconstitution, accelerating engraftment kinetics and increasing absolute cell counts across multiple immune subsets. In a therapeutic model, irradiated S15-DKO mice bearing Raji-luc cells demonstrated superior tumor reduction and significantly higher levels of cytokine release compared to DKO mice following anti-CD3xCD19 BiTE treatment.

**Discussion:**

These findings establish PBMC-humanized S15-DKO mice as a novel preclinical platform for evaluating efficacy and cytokine release of immunotherapies that require diverse populations of human immune cells.

## 1 Introduction

The successful translation of cancer immunotherapies from basic development to clinical application depends on the availability of appropriate preclinical models to predict efficacy and safety in humans. Humanized mouse models, in which immunodeficient mice are engrafted with cellular components of the human immune system, have emerged as valuable tools for evaluating and validating novel immunotherapies (1–3).

NSG-MHC I/II double-knockout (DKO) immunodeficient mice lack expression of both mouse MHC class I and class II molecules, and when engrafted with peripheral blood mononuclear cells (PBMCs) from human donors, show delayed development of xenogeneic graft-versus-host disease (xeno-GvHD) as compared to standard NSG mice (4). However, engraftment of specific human immune cell populations, such as NK cells and myeloid cells, remains limited in the DKO model (5). The absence of robust human myeloid and NK cell populations limits the use of current PBMC-based models for evaluating immunotherapies targeting these cell types. Thus, there is a critical need to develop the next generation of PBMC-humanized models that more accurately recapitulate the cellular diversity of the human immune system.

A central factor for the poor development and survival of human myeloid and NK cells in humanized mice is the species-specific incompatibility of several cytokines that regulate hematopoiesis (6, 7). Cytokines and growth factors that promote human myeloid cell development, survival, and function include stem cell factor (SCF), granulocyte-macrophage colony-stimulating factor (GM-CSF), and interleukin-3 (IL-3) (8, 9). Interleukin-15 (IL-15) is critical to the development and function of human NK cells (10). Murine versions of these cytokines exhibit limited cross-reactivity with human receptors, creating a cytokine-deficient environment that cannot support optimal human innate immune cell development. Mouse models expressing combinations of these human cytokines can support myeloid and NK cell development after engraftment with human NK cells from peripheral blood or hematopoietic stem cells (HSCs) (11–16). However, HSC engraftment models can be cost prohibitive and have longer experimental time courses than PBMC-engrafted models (17). As an alternative to HSC, the use of PBMCs for engraftment enables the rapid screening of many different donors for therapeutic efficacy. Immunodeficient mice expressing multiple human cytokines may promote a diverse repertoire of human immune cells after PBMC engraftment and broaden the application of these humanized models.

Herein, we generated a novel immunodeficient NSG mouse expressing human *KITLG* (KIT ligand; SCF), *CSF2* (colony stimulating factor 2; GM-CSF), *IL3* (interleukin 3) and *IL15* (interleukin 15) (SGM3-IL15), that does not express mouse MHC molecules (DKO). Using the NSG-SGM3-IL15-DKO (S15-DKO) mouse engrafted with PBMCs from different donors, we characterized the human immune cell engraftment profile, response to implanted human cancer cells, and immunotherapy response as compared to the DKO strain. Our data show that the S15-DKO mouse supports increased diversity of human immune cells after PBMC engraftment, enables the study of therapeutics, and displays delayed xeno-GvHD properties. Together these results demonstrate that the S15-DKO mouse is a promising preclinical model to evaluate a broad range of human specific therapeutics *in vivo*.

## 2 Materials and methods

### 2.1 Mice

Female NOD.Cg-*Prkdc^scid^ H2-K1^b-tm1Bpe^ H2-Ab1^g7-em1Mvw^ H2-D1^b-tm1Bpe^ Il2rg^tm1Wjl^* Tg(CMV-IL3,CSF2,KITLG)1Eav Tg(IL15)1Sz/J (S15-DKO, stock number #037320, expressing human *IL3*, *CSF2*, *KITLG* and *IL15* combined with mouse MHC I/II double knockout, The Jackson Laboratory, Bar Harbor, ME) and NSG-*H2-K1^b-tm1Bpe^ H2-Ab1^g7-em1Mvw^ H2-D1^b^****^-^***^tm1Bpe^ (DKO, stock number #025216, The Jackson Laboratory, Sacramento, CA) mice at 7 to 8 weeks of age were acclimated for at least 3 days after transport. Mice were housed at a density of maximum 5 mice per cage in specific pathogen-free environments, with 12-hour light/dark cycle. Standard chow and acidified water (pH 2.5-3.0) were provided *ad libitum*. Upon reaching a humane endpoint or the scheduled necropsy time point, mice were euthanized by CO_2_ asphyxiation with a chamber flow rate of 30% (VetEquip, Marsing, ID) for six minutes, followed by cervical dislocation as the secondary euthanasia method to ensure death.

### 2.2 Engraftment of mice with human PBMCs

On study day (SD) 0, S15-DKO and DKO mice received 10^7^ human PBMCs from various healthy donors (donor information including age, sex, ethnicity, vendor and the experiments is provided in **Supplementary Table 1**) via intravenous (IV) tail vein injections. For experimental designs with irradiation, body weights were matched between groups, and mice were irradiated with 100 cGy (MultiRad225, Precision X-Ray Inc, Madison, CT) at least four hours before PBMC IV injections. Body weights were recorded and clinical observations were performed for all the mice at least twice a week. GvHD symptoms were closely monitored, including hunched posture, hair loss, tachypnea and anemia. The mice were euthanized when they reached any of the following pre-defined humane endpoints: moribundity, weight loss of 20% or greater, severe hunching, severe paleness, ulcerated skin, ulcerated eye, and lethargy. A subset of mice in each group underwent necropsy on SD 21 or SD 28 to collect the spleen for evaluating engraftment. The remaining mice were followed for survival analysis. Retro-orbital (RO) blood was collected weekly (alternating eyes) into K_2_EDTA tubes to evaluate PBMC engraftment. All animal procedures were approved by the Animal Care and Use Committee of The Jackson Laboratory (IACUC #: 24-031). This study uses human PBMCs obtained from commercial vendors as listed in **Supplementary Table 1**. Pre-existing ethical approval and written informed consent were obtained by these vendors prior to sample collection. The Jackson Laboratory Institutional Review Board did not require the study to be approved by an ethics committee because this project does not meet the definition of human subjects research under Laboratory policy and applicable Federal Regulations.

### 2.3 Tumor cell lines

Early passage Raji-luc-GFP lymphoma cells (Creative Biogene Inc, Shirley, NY) were cultured in RPMI-1640 (ThermoFisher) with 10% fetal bovine serum (FBS, Fisher Scientific, Hampton, NH) and 1% penicillin-streptomycin (ThermoFisher) at 37 °C with 5% CO_2_. MDA-MB-231-luc-GFP (Cellomics Technology, Halethorpe, MD) were cultured in Leibovitz’s L-15 medium (ThermoFisher) supplemented 10% FBS, 1% penicillin-streptomycin, 1% MEM non-essential amino acids solution (NEAA, ThermoFisher), 1% L-glutamine (ThermoFisher) and 1% sodium pyruvate (ThermoFisher) at 37 °C with 0% CO_2_. Blasticidin (10 µg/mL, InvivoGen, San Diego, CA) was included in the culture media when expanding Raji-luc-GFP cells. Puromycin dihydrochloride (0.6 µg/mL, ThermoFisher) was added when expanding MDA-MB-231-luc-GFP cells. Blasticidin and puromycin dihydrochloride were removed for the last passage before injection into the mice.

### 2.4 Tumor xenograft models

Raji-luc-GFP cells (2.5x10^5^ cells/mouse, ≤10 passages) were injected IV into mice five days before PBMC injection (SD -5). MDA-MB-231-luc cells (5.0x10^5^ cells/mouse, ≤10 passages) were injected IV two days before PBMC injection (SD -2). PBMCs (1.5 x10^7^ per mouse) were injected IV on SD 0. Around half of the mice in the PBS and Raji-luc-GFP group were taken down on SD 14. Tumor burdens were measured by *in vivo* bioluminescent imaging using the Lumina IVIS^®^ S5 Imaging System (PerkinElmer, Springfield, IL). D-luciferin (150 mg/kg in PBS, PerkinElmer, Springfield, IL) was intraperitoneally (IP) injected, and mice were imaged around 10 minutes later. The mice were maintained under 1-2% isoflurane anesthesia during ventral and dorsal imaging (auto exposure). The region of interest in the analysis included the whole body except for the tail. The bioluminescence is presented as total flux (photons/second [p/s]).

To test the efficacy and toxicity of BiTEs, 1.5 x10^7^ PBMCs were injected IV after irradiation on SD 0, followed by IV injection of 4 x10^6^ Raji-luc-GFP cells per mouse on SD 5. On SD 6, PBS or anti-CD3xCD19 BiTE (0.1 mg/kg, SinoBiological, Houston, TX) were delivered IV to the Raji-luc cell-bearing mice. Six hours later, RO blood was collected for plasma. Tumor burdens were measured by bioluminescent imaging on SD 6, SD 8 and SD 11 to check the efficacy of anti-CD3xCD19 BiTE. Cytokine levels were measured with BD cytometric bead array (CBA) human Th1/Th2 kit (BD Bioscience, San Diego, CA) or the 48-plex MILLIPLEX Human Cytokine/Chemokine/Growth Factor Panel A premixed magnetic bead panel (HCYTA-60K-PX48, Millipore, Burlington, MA) following the manufacturer’s instructions.

### 2.5 Spleen cell processing for flow cytometry

Spleens were harvested in MACS separation buffer (Miltenyi Biotec, San Diego, CA) with 0.5% BSA (MAG buffer) on SD 21 or SD 28 or end of life and crushed with a syringe plunger onto a 70 µm cell strainer on a 6-well plate. Cell strainers were rinsed with MAG buffer, and the final volume was brought to 3–5 ml depending on the spleen size. The cell suspension was transferred to a 40 µm cell strainer on a 25 ml canonical tube to further remove any potential clumps prior to flow cytometry staining.

### 2.6 Flow cytometry analysis

To evaluate immune cell types and engraftment levels, RO blood and spleen single-cell suspension samples were incubated with anti-mouse CD16/CD32 (mouse Fc block, Clone 2.4G2, BD Bioscience) and antibodies for 30 minutes at room temperature, then 1x ACK Lysing Buffer (ThermoFisher) was added for 10 minutes to remove red blood cells. The cells were centrifuged and resuspended in 1x ACK Lysing Buffer. They were centrifuged and resuspended in PBS + 1% FBS. CountBright Absolute Counting Beads (ThermoFisher) were added to all the samples, and they were analyzed on a FACSymphony A5 Cell Analyzer (BD). The following antibodies were used in the panel: Spark UV387 anti-human HLA-DR (Clone L243, BioLegend, San Diego, CA), Brilliant Ultraviolet 737 anti-human CD19 (Clone SJ25C1, Invitrogen, Carlsbad, CA), Brilliant Violet 421 anti-human CD8a (Clone RPA-T8, BioLegend), Brilliant Violet 510 anti-human CD3 (Clone UCHT1, BioLegend), Brilliant Violet 605 anti-human CD4 (Clone SK3, BioLegend), Brilliant Violet 650 anti-mouse CD45 (Clone 30-F11, BioLegend), BV786 anti-human CD56 (Clone NCAM16.2, BD Biosciences), 7-AAD viability staining solution (BioLegend), PE/Dazzle™ 594 anti-human CD64 (Clone 10.1, BioLegend), PE/Cyanine7 anti-human CD11c (Clone S-HCL-3, BioLegend) and Alexa Fluor^®^ 700 anti-human CD45 (Clone HI30, BioLegend).

### 2.7 Human IgG ELISA measurement

Plasma collected from cardiac puncture blood on SD 28 in S15-DKO and DKO mice engrafted with PBMCs from donor 1309 was used to measure human IgG following the instructions of the ELISA kit from Biorbyt (Durhan, NC).

### 2.8 Histology, immunohistochemistry, and pathology

Tissues were fixed in 10% neutral buffered formalin for 48 h, transferred to 70% ethanol, embedded in paraffin, and sectioned at 5 μm. Slides were stained with hematoxylin and eosin (H&E) or used for immunohistochemistry (IHC). For IHC, tissue sections were mounted on ColorFrost™ Plus Microscope Slides (Fisherbrand 12-550-17). The IHC procedure was performed using a Leica BOND RX automated platform. Briefly, after dewaxing and rehydration, sections were pretreated with BOND-PRIME Epitope Retrieval Solution 1 (Leica AR0086) for 20 minutes at 98 °C. Endogenous peroxidase was inactivated with 3% hydrogen peroxide for 10 minutes at room temperature. Nonspecific tissue-antibody interactions were blocked with Leica PowerVision IHC/ISH Super Blocking solution (PV6122) for 30 minutes at room temperature. The same blocking solution also served as diluent for the primary anti-CD11c antibody (Abcam #ab52632; 1:1000). Bond Polymer Refine Detection (Leica DS9800) was used for chromogenic visualization. Slides were counterstained in hematoxylin, dehydrated in an ethanol series, cleared in xylene, and coverslipped. Slides were qualitatively evaluated by an ACVP board-certified veterinary pathologist using an Olympus BX43 brightfield light microscope (Olympus, Tokyo, Japan). Photomicrographs were captured with an Olympus DP74 camera and cellSens Standard 3.2 software (Olympus, Tokyo, Japan).

### 2.9 Statistical analysis

Data are presented as mean ± SEM. Log-rank tests were conducted on survival data. Mann-Whitney tests were performed on scatter plots to compare engraftment in S15-DKO and DKO mice. One-way or two-way ANOVA analysis (full model) with Sidak’s multiple comparison within each row was used to analyze time course data and data involving Raji-luc-GFP cells. Statistical analysis was performed with GraphPad Prism 10.3.

## 3 Results

### 3.1 Increased PBMC engraftment in S15-DKO mice

S15-DKO and DKO mice were injected IV with human PBMCs (1x10^7^ cells) from four different donors (1309, 1610, 5822, and 6184). Body weight (BW), survival and human CD45 engraftment were monitored (**Figure 1**). Starting BW varied across donors. BW increased steadily for both strains in donors 1309, 1610 and 6184 (**Figure 1A**). In donor 5822, BW of S15-DKO mice remained relatively stable (23 to 24 g), whereas BW of DKO increased steadily over the course of the experiment (19 g to 22.6 g). Mild GvHD symptoms, including rough/scruffy fur and slight hair loss on back, are commonly observed in both strains following PBMC engraftment. Skin lesions may appear in both S15-DKO mice and DKO mice approximately 6 to 7 weeks after PBMC engraftment. Overall survival of PBMC-engrafted S15-DKO and DKO mice was similar for each PBMC donor with minimal mortality through the end of the study (35 to 91 days) (**Figure 1B**). Flow cytometry gating strategies are shown in **Supplementary Figure 1A**. Human PBMCs from four different donors showed different engraftment profiles in both S15-DKO and DKO mice (**Figures 1C, D**). PBMCs engrafted faster in S15-DKO mice compared to DKO mice; the peak engraftment of human CD45^+^ cells was on SD 28 or SD 35 (depending on the donor) in S15-DKO mice, whereas the peak engraftment occurred 7 days later in DKO mice engrafted with PBMCs from the same donor (**Figure 1D**). In addition, for each donor, the human CD45^+^ cell peak engraftment percentage (**Figure 1C**) and absolute human CD45^+^ cell count (**Figure 1D**) in whole blood of S15-DKO mice were higher than those in whole blood of DKO mice. H&E staining revealed mononuclear cell infiltration in the portal regions of the liver in both S15-DKO and DKO mice engrafted with donor 1309 on SD 28. No significant difference was observed between the two strains, as shown in **Supplementary Figure 1B**. The steady BW increase, limited mortality and the histopathology findings from H&E staining in engrafted mice prove the delayed xeno-GvHD in the S15-DKO strain. Notably, PBMCs from donor 1610 were engrafted at a low level in DKO mice until SD 35, so this donor was not followed for a longer period.

**Figure 1:**
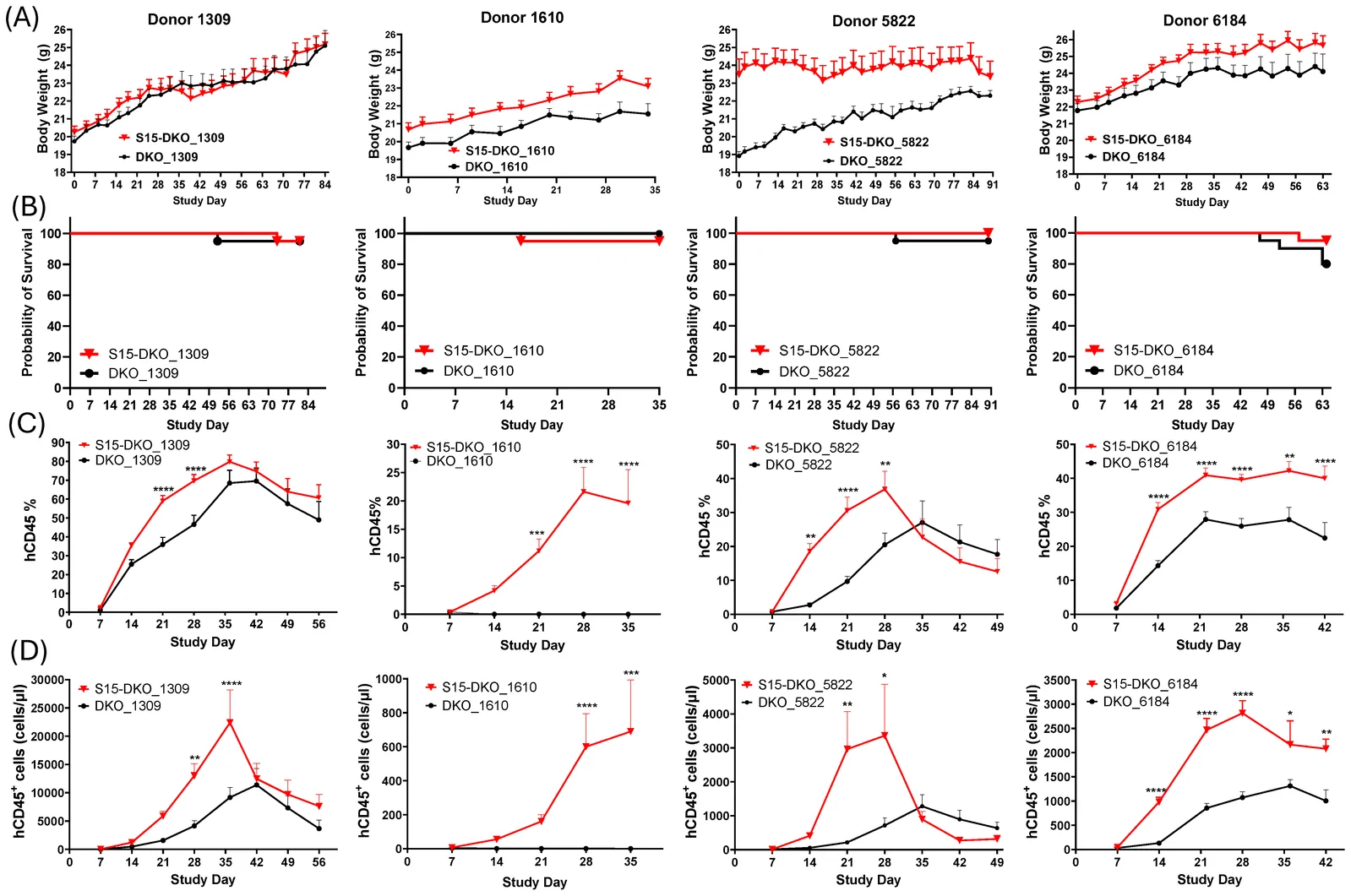
Body weight, survival and human CD45^+^ cell engraftment in S15-DKO and DKO mice. **(A)** Body weight, and **(B)** survival of non-irradiated S15-DKO and DKO mice (N = 20, 10 were euthanized on SD 28 to collect spleen data) after engraftment of PBMCs (IV injection of 1x10^7^ cells) from donors (columns from left to right) 1309, 1610, 5822 and 6184. **(C)** Percentage and **(D)** absolute cell count of human CD45^+^ cells in the S15-DKO (red inverted triangles) and DKO (black circles) mice over time. Data are presented as mean ± SEM (n=20 by study day 28), Log-rank test was conducted on survival data. Two-way ANOVA analysis (full model) with Sidak’s multiple comparison within each row was conducted in **(C–D)**. * *p* < 0.05, ** *p* < 0.01, *** *p* < 0.001 and **** *p* < 0.0001.

For each of three PBMC donors (1309, 5822, and 6184), CD4^+^ and CD8^+^ T cells (**Figure 2A**), CD19^+^ B cells (**Figure 2B**; donor 1309 and 6184), and CD56^+^ NK cells (**Figure 2C**) in the blood showed significantly higher peak engraftment levels in S15-DKO mice compared to DKO mice. Representative flow cytograms of the above cell populations are shown in **Supplementary Figure 2A**-2D. We also use expression of HLA-DR (an acute activation marker on T cells (18)) to evaluate T cell activation in the blood for at least 42 days. The frequency of CD4^+^HLA-DR^+^ cells and CD8^+^HLA-DR^+^ cells of S15-DKO mice was similar to or lower than in DKO mice engrafted with donor 1309, 5822 and 6184 (**Supplementary Figures 2F–I**), suggesting that T cells expansion in S15-DKO mice reflects useful immune reconstitution without excessive activation. On SD 28, the number of human CD19^+^ B cells in the S15-DKO mouse spleens were significantly higher than in DKO mouse spleens for two of three donors (**Figure 2D**). Human CD56^+^ NK cell counts were significantly higher in S15-DKO spleens compared to those from DKO mice (**Figure 2E**). We normally observe very few, if any, human dendritic cells (DC, HLA-DR^+^CD11c^+^) engrafted in DKO mice, and we found DCs tend to engraft in more of the S15-DKO mice than DKO mice (**Figure 2F**). Furthermore, for donor 6184-engrafted mice, we found significantly higher levels of dendritic cells (HLA-DR^+^CD11c^+^) and macrophage (HLA-DR^+^CD64^+^) engraftment in S15-DKO than DKO mice (**Figure 2F**). Representative flow cytograms of CD3^-^CD19^-^HLA-DR^+^CD64^+^ macrophages and CD3^-^CD19^-^HLA-DR^+^CD11c^+^ dendritic cells are presented in **Supplementary Figure 2E**. In anti-human CD11c IHC staining of livers from S15-DKO and DKO mice engrafted with donor 1309 on SD 28, low numbers of mononuclear cells displayed cytoplasmic immunoreactivity to anti-CD11c, indicating human dendritic cell origin (**Supplementary Figure 2J**).

**Figure 2:**
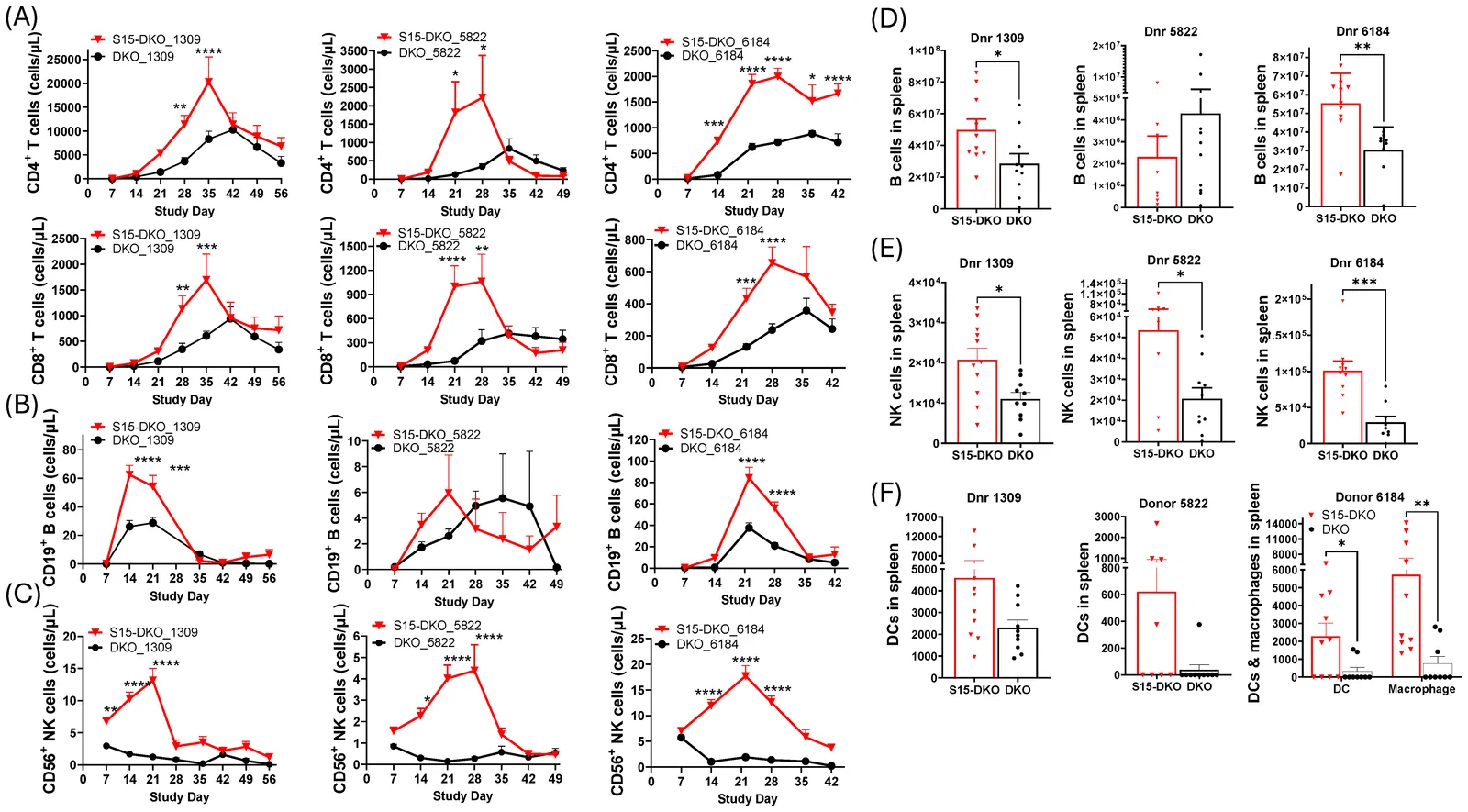
Engraftment of human immune cell populations in the blood and spleen of S15-DKO and DKO mice. Absolute cell counts of **(A)** human CD4^+^ T cells and CD8^+^ T cells, **(B)** human CD19^+^ B cells, and **(C)** human CD56^+^ NK cells in whole blood of non-irradiated S15-DKO (red inverted triangles) and DKO (black circles) mice engrafted with PBMCs from donor 1309 (left), 5822 (middle) or 6184 (right). Absolute cell counts of **(D)** human CD19^+^ B cells, **(E)** human CD56^+^ NK cells, and **(F)** human CD3^-^CD19^-^HLA-DR^+^CD11c^+^ dendritic cells (DCs) and CD3^-^CD19^-^HLA-DR^+^CD64^+^ macrophages in the spleens of S15-DKO (red inverted triangles) and DKO (black circles) mice on SD 28. Data are presented as mean ± SEM (n=20 by study day 28). Two-way ANOVA analysis (full model) with Sidak’s multiple comparison within each row was conducted in **(A–C)**, Mann-Whitney test was performed in **(D–F)**, * *p* < 0.05, ** *p* < 0.01, *** *p* < 0.001 and **** *p* < 0.0001.

We engrafted PBMCs from five additional donors (0952, 2870, 3161, 2786, and 3643) and observed steady body weight increase and robust survival of S15-DKO and DKO mice until the end of the study on SD 84 (**Supplementary Figures 3A, B**), similar to **Figures 1A, B**. We also observed higher levels of human CD45^+^ cells in S15-DKO spleens on SD 21 compared to DKO spleens (**Supplementary Figure 3C**, significantly higher for three out of five donors), similar to **Figure 1D**. In addition, there were significantly higher numbers of CD19^+^ B cells (three out of five donors, **Supplementary Figure 3D**) and CD19^+^CD138^+^ plasma cells (majority of these cells are also CD27^+^) in the spleens from S15-DKO mice compared to DKO mice on SD 21 (**Supplementary Figure 3E**). Total human IgG from S15-DKO mice was significantly higher than DKO mice on SD 28 with donor 1309 (**Supplementary Figure 3F**), which indicates higher engraftment level of plasma cells, as we observed in other donors. A summary of B cell engraftment across the ten PBMC donors used in the mice is presented in **Supplementary Table 2**.

### 3.2 Lymphoma boosted PBMC engraftment of DC, NK and T cells in S15-DKO mice

Next, we evaluated the humanized immune response of PBMC-engrafted non-irradiated S15-DKO and DKO mice to cancer cell exposure. When the mice were implanted with Raji lymphoma cells on SD -5 before PBMC engraftment, we observed a significantly higher human CD45^+^ cell count in both S15-DKO and DKO mice on SD 10 compared to controls receiving only PBS on SD -5 (**Figure 3A**). Significantly higher numbers of human CD4^+^ T cells, human CD56^+^ NK cells and human CD3^-^CD19^-^HLA-DR^+^CD11C^+^ DCs were seen in the blood from S15-DKO mice compared to DKO mice on SD 10 (**Figure 3A**). By SD 21, Raji cells were eliminated in both S15-DKO and DKO mice because of an allogeneic specific response mediated by the PBMCs (data not shown). In the groups without implanted Raji lymphoma cells, S15-DKO mice still had significantly higher numbers of human CD4^+^ T cells, B cells and DCs compared to DKO mice (**Figure 3B**).

**Figure 3:**
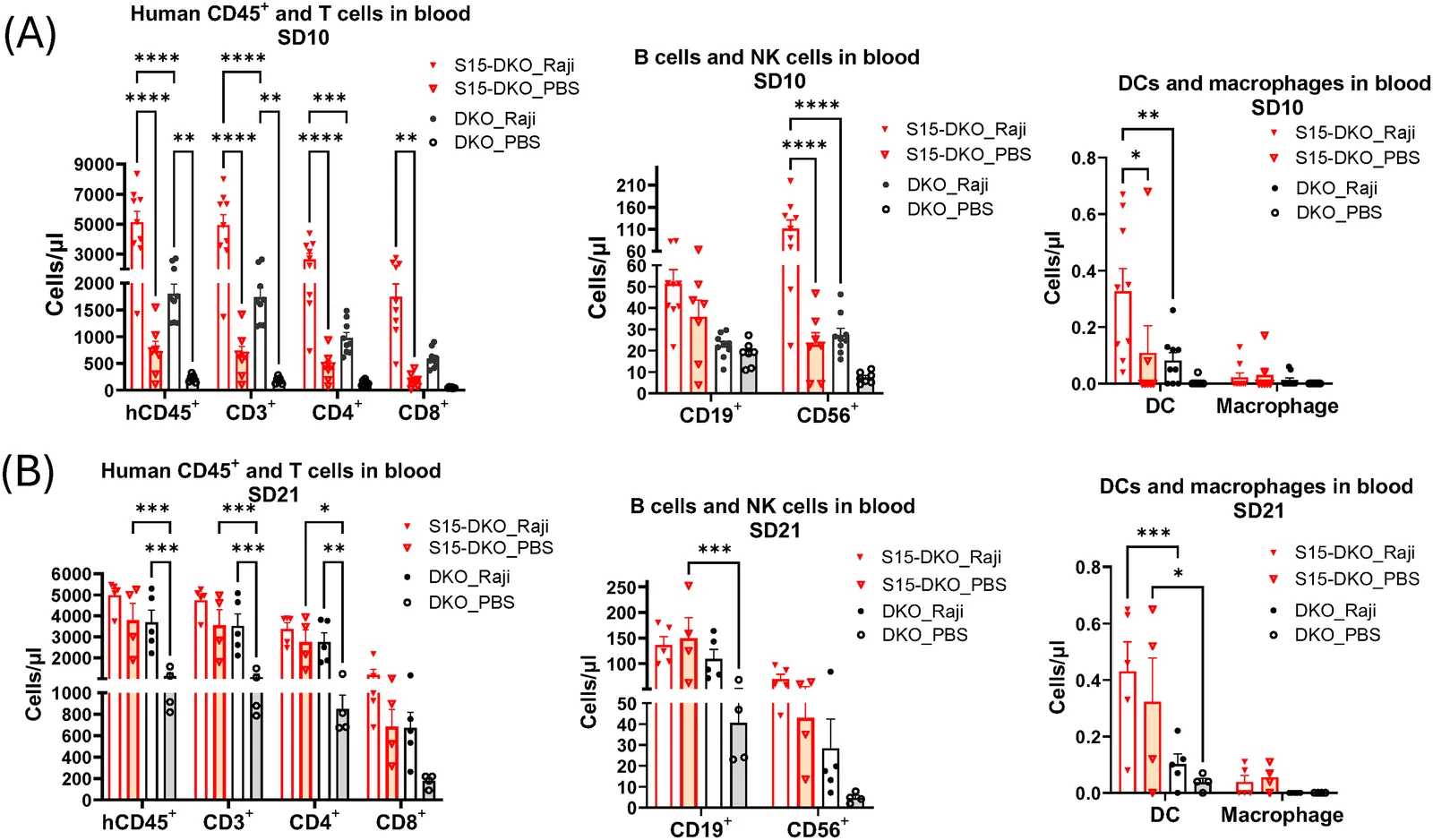
Human PBMC engraftment in S15-DKO and DKO mice implanted with Raji lymphoma cells. Absolute cell counts of human CD45^+^ cells, CD3^+^ T cells, CD4^+^ and CD8^+^ T cells, CD19^+^ B cells and CD56^+^ NK cells, and CD3^-^CD19^-^HLA-DR^+^CD11C^+^ DCs and CD3^-^CD19^-^HLA-DR^+^CD64^+^ macrophages on **(A)** SD 10 and **(B)** SD 21 in S15-DKO (red inverted triangles) and DKO (black circles) mice injected with PBS (unfilled symbols, N = 7 on SD 10, and N = 4 on SD 21 (three mice were scheduled to be taken down on SD 14)) or Raji cells (filled symbols, N = 9 on SD 10 and N = 5 on SD 21 (four mice were scheduled to be taken down on SD 14)) 5 days before injection with 1.5x10^7^ PBMCs from donor 6184. Data are presented as mean ± SEM; significance was evaluated with two-way ANOVA (full model) with Sidak’s multiple comparison within each row, * *p* < 0.05, ** *p* < 0.01, *** *p* < 0.001 and **** *p* < 0.0001.

### 3.3 Irradiation enhances PBMC engraftment in S15-DKO mice

PBMC engraftment in immunodeficient mice is enhanced significantly by preconditioning of recipients with irradiation (19). We next compared engraftment of human PBMC in irradiated (100 cGy) or non-irradiated S15-DKO mice. PBMC donor 0877 showed no significant differences in BW gain or survival between irradiated and non-irradiated S15-DKO mice (**Figures 4A, B**). In contrast, irradiation caused significant BW loss immediately after irradiation, slower BW gain, and slightly higher mortality (not statistically significant) in S15-DKO mice engrafted with PBMC donor 8094, compared to non-irradiated S15-DKO mice. These results suggest that the effect of irradiation on xeno-GvHD severity may be donor-dependent. Irradiation accelerated human CD45^+^ cell engraftment kinetics, shifting the peak percentages of CD45^+^ cells to within approximately the first two weeks after PBMC injection (**Figure 4C**). Irradiation may also increase the peak CD45^+^ cell number depending on the PBMC donor (**Figure 4D**). At early time points post-engraftment, irradiation increased the levels of human CD3^+^ T cells (mainly CD4^+^ T cells), CD19^+^ B cells, and CD56^+^ NK cells in S15-DKO mice (**Figures 4E, F**). More importantly, irradiation markedly increased engraftment of B cells (**Figure 4G**) and NK cells (**Figure 4H**) in S15-DKO mice, which are notoriously difficult to engraft in PBMC-humanized mouse models. Donor 8094 was engrafted with higher immune cell counts compared to donor 0877, which may account for the BW and mortality difference between these two donors we observed in **Figures 4A, B**. In addition, we observed significantly higher levels of IFN-γ (two donors) and IL-2 (donor 1149) in irradiated S15-DKO mice compared to non-irradiated S15-DKO mice (**Figure 4I**) on SD 6 post-PBMC engraftment. More irradiated S15-DKO mice also had measurable levels of IL-10 compared to non-irradiated S15-DKOs.

**Figure 4:**
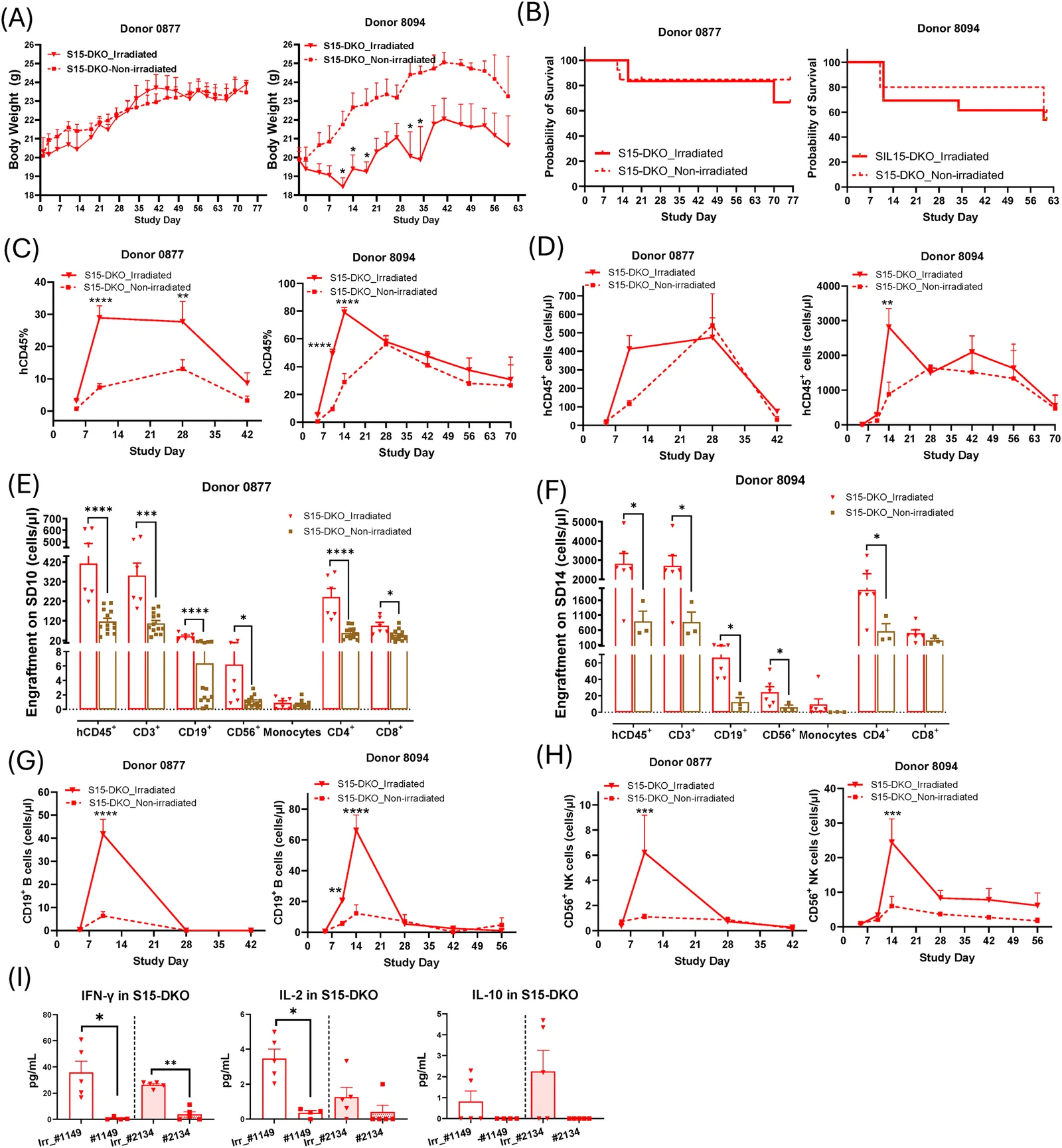
Irradiation increases PBMC engraftment and cytokine release in S15-DKO mice. **(A)** Body weight changes and **(B)** survival of S15-DKO mice engrafted with donor 0877 (N = 6 with irradiation and N = 13 without irradiation) or donor 8094 (N = 13 with irradiation and N = 12 without irradiation) with (inverted triangles) or without (squares) irradiation prior to PBMC injection. **(C)** Percentage and **(D)** cell count of human CD45^+^ cells in whole blood of S15-DKO mice with (inverted triangles) or without (squares) irradiation. Immune cell counts in whole blood of mice receiving PBMCs from **(E)** donor 0877 (SD 10) and **(F)** donor 8094 (SD 14). Whole blood cell counts of **(G)** CD19^+^ B cells, and **(H)** CD56^+^ NK cells across time for mice engrafted with PBMCs from donors 0877 (left) and 8094 (right). **(I)** Plasma interferon (IFN)-γ, interleukin (IL)-2 and IL-10 on SD 6 in irradiated (irr) and non-irradiated S15-DKO mice engrafted with donor 1149 (blank) and 2134 (red) bearing 2x10^6^ Raji-luc cells implanted on SD 5. Data are presented as mean ± SEM. Two-way ANOVA analysis (full model) with Sidak’s multiple comparison within each row was performed, * *p* < 0.05, ** *p* < 0.01, *** *p* < 0.001 and **** *p* < 0.0001.

When S15-DKO and DKO mice were irradiated before PBMC engraftment, both strains showed a steady BW increase in mice engrafted with donor 0877 (**Figure 5A**). However, mice engrafted with donor 8094 showed a mild BW drop at early timepoints, particularly in S15-DKO mice, before BW recovered and then increased. Both mouse strains had high survival to at least SD 28 (**Figure 5B**). Compared to non-irradiated mice (**Figure 1B**), both irradiated S15-DKO and DKO mice showed higher mortality levels. Irradiated S15-DKO mice had a higher percentage and cell count of human CD45^+^ cells in whole blood compared to irradiated DKO mice (**Figure 5C**). In both donors, time to reach the highest engraftment of human CD45^+^ cell counts was different in S15-DKO mice compared to DKO mice, showing different engraftment kinetics after irradiation. The peak level of CD4^+^ T cells was higher in irradiated S15-DKO compared to irradiated DKO mice (**Figure 5D**). Irradiated S15-DKO showed a significantly higher engraftment in one of the two donors of CD8^+^ T cells (**Figure 5E**; donor 0877), CD19^+^ B cells (**Figure 5F**; donor 8094) and CD56^+^ NK cells (**Figure 5G**; donor 0877) compared to irradiated DKO mice.

**Figure 5:**
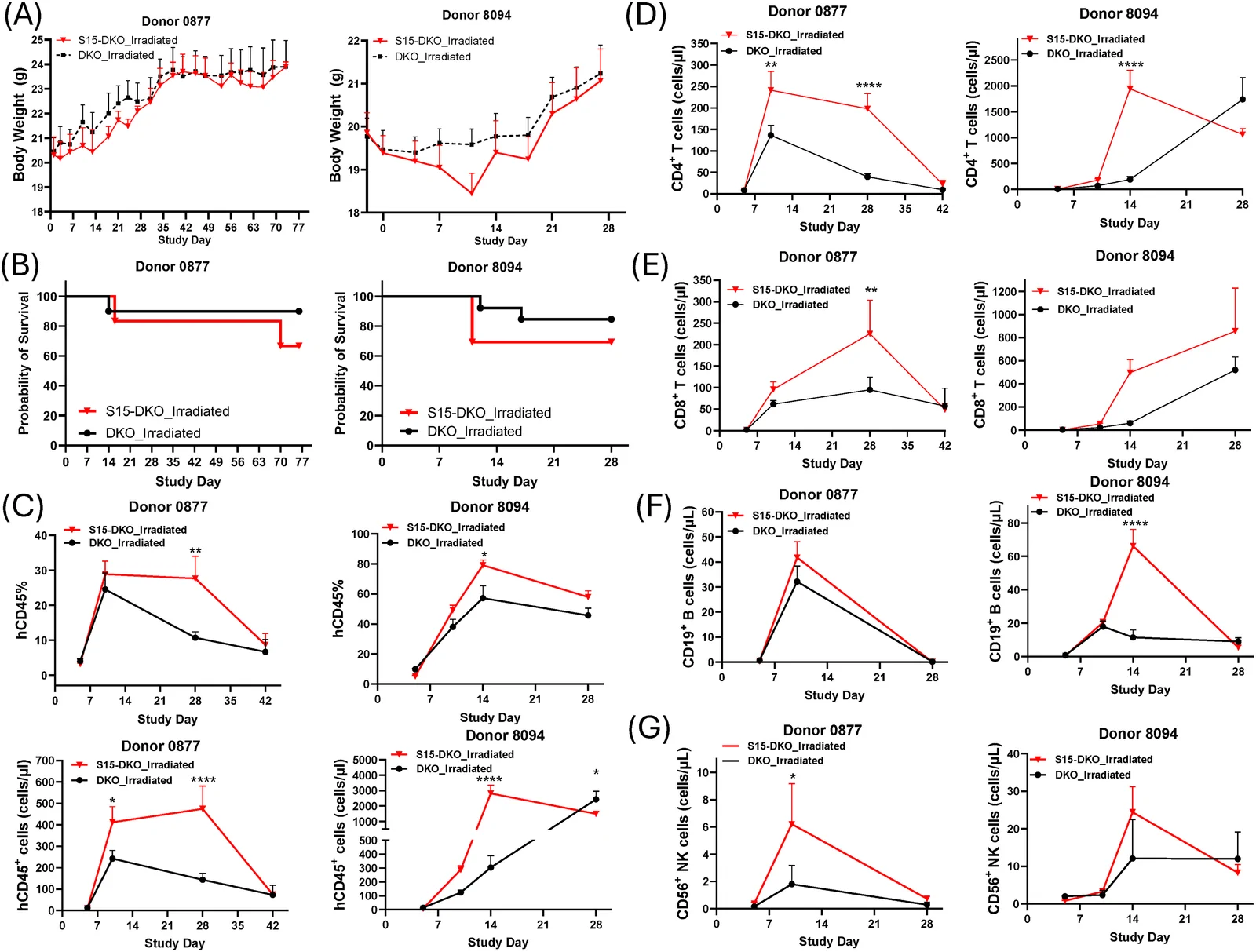
Body weight, survival and immune cell engraftment in irradiated S15-DKO and DKO mice. **(A)** Body weight and **(B)** survival of irradiated S15-DKO (red inverted triangle) and DKO (black square) mice that were irradiated and engrafted with PBMCs from donor 0877 (left, N = 6 S15-DKO mice and N = 10 DKO mice) and 8094 (right, N = 13 of both strains). **(C)** Percentage and cell count of human CD45^+^ cells in irradiated S15-DKO and DKO mice. Absolute cell counts of **(D)** CD4^+^ T cells, **(E)** CD8^+^ T cells, **(F)** CD19^+^ B cells and **(G)** CD56^+^ NK cells in irradiated S15-DKO and DKO mice. Data are presented as mean ± SEM. Two-way ANOVA analysis (full model) with Sidak’s multiple comparison within each row was performed, * *p* < 0.05, ** *p* < 0.01, *** *p* < 0.001 and **** *p* < 0.0001.

We then evaluated the response to cancer cell exposure after irradiation. The irradiated, PBMC-engrafted S15-DKO mice showed a stronger humanized immune response to the Raji cells compared to irradiated, PBMC-engrafted DKO mice (**Supplementary Figures 4A–G**). Therefore, Raji-luc cells were cleared out significantly faster in S15-DKO mice compared to DKO mice up to SD 8 because of an allogeneic response from the PBMCs (**Supplementary Figure 4H**). A similar effect was observed after exposure to MDA-MB-231-luc breast cancer cells, though both the irradiated, PBMC-engrafted S15-DKO and DKO mice had reduced responses to the MDA-MB-231-luc cells compared to the Raji-luc cells. Unsurprisingly, MDA-MB-231-luc cells did not expand as quickly in S15-DKO mice compared to in DKO mice (**Supplementary Figure 4I**).

### 3.4 Robust efficacy and cytokine release of BiTEs in S15-DKO compared to DKO mice

BiTEs are immunotherapeutics that guide T cells to the tumor-specific antigens, thus triggering cytotoxic T cell responses and inducing tumor cell death (20). The anti-CD3xCD19 BiTE was approved for treatment of acute lymphoblastic leukemia (21). Cytokine release syndrome (CRS) is an acute toxicity that has been a challenge for BiTE development and clinical use (22), and therefore preclinical models are needed to identify candidate therapeutics with reduced toxicity (23). Our pilot study results show that irradiation enables a broad cytokine response and is needed for robust cytokine production with different donors in PBMC-humanized DKO mice treated with BiTEs (**Supplementary Figure 5**).

Irradiated PBMC-engrafted S15-DKO and DKO mice bearing Raji-luc cells were then treated with anti-CD3xCD19 BiTE or PBS. In mice engrafted with donor 8130, anti-CD3xCD19 BiTE treatment significantly reduced tumor burden in S15-DKO but not DKO mice on SD 8, though significant tumor reduction was observed in both strains by SD 11 (**Figure 6A**). In mice engrafted with donor 9585, anti-CD3xCD19 BiTE treatment significantly decreased tumor burden in both Raji-bearing S15-DKO and DKO mice on SD 8. Tumor burden also declined in PBS-treated mice engrafted with both PBMC donors by SD 11 because of an allogeneic response. Therefore, the decreased tumor burden in the anti-CD3xCD19 BiTE groups reflects a combined effect of alloreactivity from engrafted PBMC immune cells and BiTE-specific activity.

**Figure 6:**
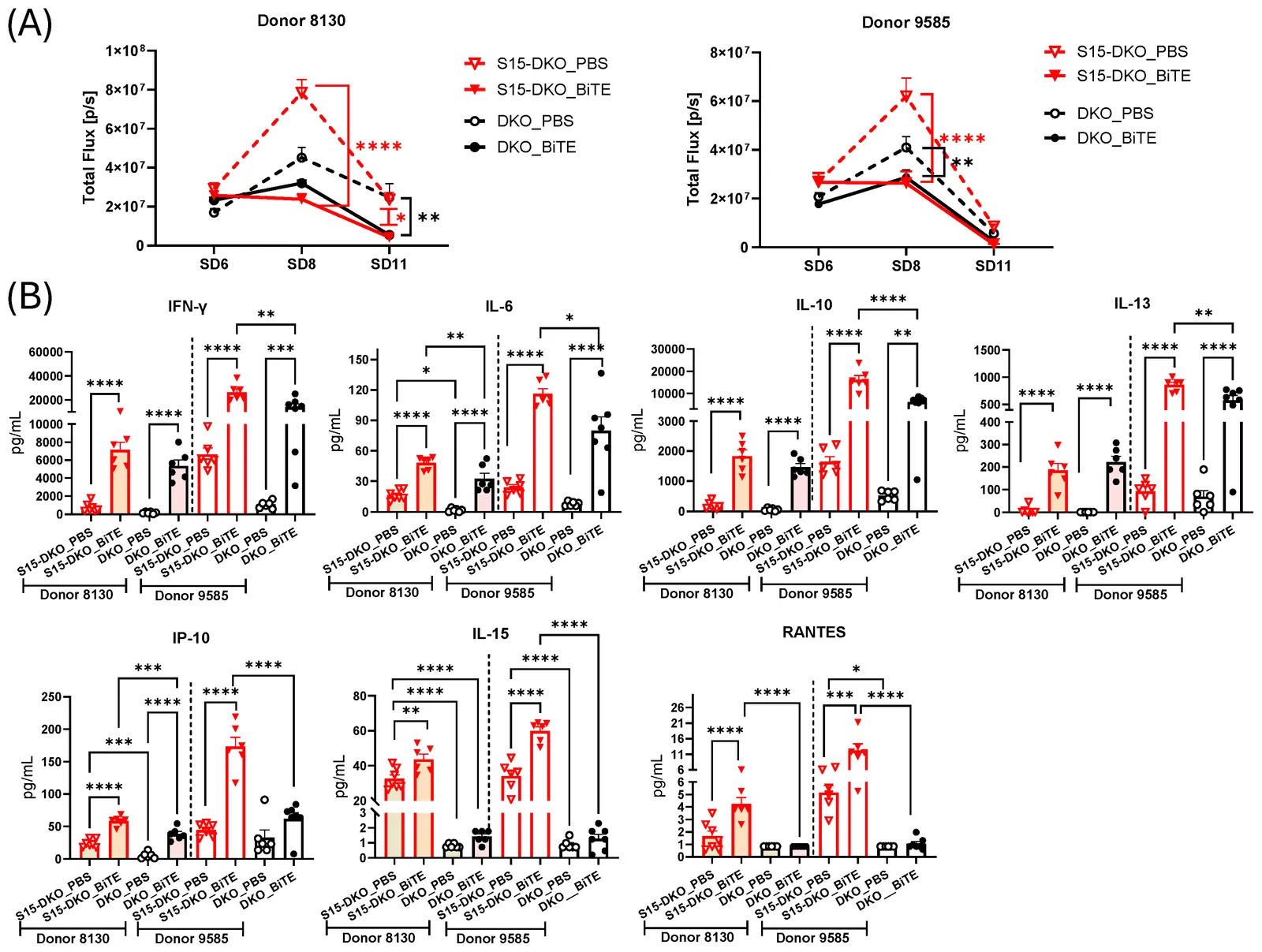
Effects of anti-CD3xCD19 BiTE treatment on tumor burden and cytokine production in S15-DKO and DKO mice. **(A)** Tumor burden of Raji lymphoma cells on SD 6 (pre-treatment), SD 8 and SD 11 in irradiated S15-DKO (red inverted triangles) or DKO (black circles) mice after PBS (unfilled symbols) or anti-CD3xCD19 BiTE treatment (filled symbols). Mice were PBMC-humanized with donor 8130 (left, N = 7 in PBS group and N = 6 in BiTE group of S15-DKO, N = 6 in both PBS and BiTE groups of DKO) or 9585 (right, N = 6 in both PBS and BiTE groups of S15-DKO, N = 6 in PBS group and N = 7 in BiTE group of DKO). **(B)** Plasma cytokine levels (pg/ml) of IFN-γ, IL-6, IL-10, IL-13, interferon gamma-induced protein 10 (IP-10), IL-15, and RANTES at 6 hours after PBS or anti-CD3xCD19 BiTE treatment. Data are shown as mean ± SEM. One-way ANOVA analysis with multiple comparisons, * *p* < 0.05, ** *p* < 0.01, *** *p* < 0.001 and **** *p* < 0.0001.

We also measured anti-CD3xCD19 BiTE-induced cytokine release in the Raji-bearing S15-DKO and DKO mice. In both Raji-bearing S15-DKO and DKO mice, anti-CD3xCD19 BiTE induced IFN-γ, IL-6, IL-10, IL-13 and interferon gamma-induced protein 10 (IP-10) (**Figure 6B**). In addition, anti-CD3xCD19 BiTE treatment induced IL-15 and RANTES responses in Raji-bearing S15-DKO but not DKO mice (**Figure 6B**). Notably, donor 9585-engrafted S15-DKO mice showed significantly higher levels of IFN-γ, IL-6, IL-10, IL-13, IL-15, RANTES and IP-10 compared to DKO mice after anti-CD3xCD19 BiTE treatment. In donor 8130-engrafted S15-DKO mice, IL-6, IL-15 and IP-10 were higher than in DKO mice in both the PBS and anti-CD3xCD19 BiTE treatment groups. The levels of other cytokines are presented in **Supplementary Figure 6**. In addition to IL-15, we observed significantly higher levels of GM-CSF and IL-3 in PBS-treated S15-DKO mice compared to DKO mice, due to expression of human *CSF2* and *IL3* in S15-DKO (**Supplementary Figure 6A**). Chemokine/cytokines that are produced by macrophages and dendritic cells, like MDC and MIG/CXCL9 showed higher levels in the PBS-treated S15-DKO compared to DKO (**Supplementary Figure 6B**), which supports higher engraftment cell counts of dendritic cells and macrophages in certain donors we observed in S15-DKO mice (**Figure 2F**).

## 4 Discussion

In this study, we developed and characterized the novel S15-DKO humanized mouse model, which combines the delayed xeno-GvHD properties of NSG-MHC I/II DKO mice with the human immune diversity of NSG-SGM3-IL15 mice. Our results demonstrate robust engraftment of human donor PBMCs in S15-DKO mice, which is enhanced with irradiation. S15-DKO mice show higher engraftment levels of multiple human immune cell populations compared to DKO mice, including T cells and NK cells across all donors we tested, and B cells, dendritic cells, and macrophages in some PBMC donors. Furthermore, S15-DKO mice demonstrate superior sensitivity for evaluating the efficacy and cytokine release of BiTEs in a tumor xenograft model. We also show that delayed xeno-GvHD is similar for both S15-DKO and DKO mice. The results were largely consistent across multiple PBMC donors, though there was variability in the engraftment levels. Overall, these findings establish PBMC-humanized S15-DKO mice as an improved preclinical platform for immunotherapy development that more accurately recapitulates the cellular diversity of the human immune system than PBMC-humanized DKO mouse models.

The improved NK cell engraftment in PBMC-humanized S15-DKO mice addresses a limitation of previous humanized mouse models. NK cells play essential roles in tumor immunosurveillance and responses to various immunotherapies, yet they have been difficult to establish in PBMC-humanized mice (1, 4). The consistently high NK cell numbers we observed across multiple donors and timepoints suggest that S15-DKO mice can support a NK cell compartment from peripheral blood-derived cells. This is consistent with previous reports that human IL-15, GM-CSF and IL-3 provide supportive environment for NK cells to survive and proliferate through direct and indirect interactions (8, 14, 15, 24). Higher engraftment of NK cells in S15-DKO mice after exposure to Raji-luc cells on SD 10 shows that these NK cells are potentially functional and can respond to allogeneic lymphoma. Further studies are needed to demonstrate that these engrafted NK cells are indeed functional. If confirmed, this capability will be particularly valuable for evaluating immunotherapies, especially NK cell engagers, checkpoint inhibitors that enhance NK cell function, and CAR-NK cells (25, 26).

The enhanced B cell engraftment observed in S15-DKO mice with six out of ten PBMC donors represents an advancement for modeling humoral immunity (4). We observed higher numbers of CD19^+^ B cells in the peripheral blood and spleen of PBMC-engrafted S15-DKO mice than in DKO mice in the six donors. The increased B cell engraftment in some donors may result from human *IL15* expression in S15-DKO mice, T-cell-dependent activation, and T-cell-independent B cell activation in an enhanced cytokine environment. Firstly, in addition to supporting the survival and activation of NK cells, NKT cells and CD8^+^ memory T cells (27), IL-15 is able to induce proliferation and differentiation of activated B cells *in vitro*, with activity comparable to IL-2 (28). IL-15-induced proliferation of activated B cells relies on IL2/15Rβ (28). In addition, monocytes/macrophages can trans-present IL-15 to resting B cells and cooperate with T-cell-derived CD40L to promote B cell proliferation through STAT5 activation (29). Dendritic cells may also trans-present IL-15 to germinal center B cells to enhance B cell proliferation via cell-cell contact (30), although we have not observed structured lymphoid architectures in S15-DKO mice (data not shown). Secondly, higher T cell engraftment in S15-DKO mice may promote B cell maturation through direct cell interactions, as *in vivo* T cell depletion decreases the percentage of mature B cells (31). Thirdly, the enhanced cytokines environment in S15-DKO mice, including GM-CSF, IFN-γ and IL-10, may promote B-cell-activating factor (BAFF) secretion (32, 33). Furthermore, we found the CD19^+^CD138^+^ plasma cell population (most of which is also CD27^+^, data not shown) was elevated in S15-DKO spleens compared to DKO mice. Although transcription factors or cytokines known to support B cell survival, maturation and humoral immune responses are not transgenically expressed in S15-DKO mice, we anticipate that these plasma cells are functional, as a significantly higher level of total human IgG was observed in the S15-DKO mice compared to DKO mice. In addition, our team has shown that antigen-specific human IgG antibodies were produced after Tdap or COVID-19 mRNA vaccines were given to S15-DKO mice after irradiation (34). With H5N1 vaccine, we may have also observed a naïve B cell response from human B cells in S15-DKO mice (34). In NSG mice transgenically expressing *KITLG*, *CSF2* and *IL3* (NSG-SGM3) engrafted with hematopoietic stem cells, autologous fetal liver and thymic tissues, B cell development was improved, and antigen-specific IgM and IgG were generated against dengue virus infection (35). IL-15 has been reported to induce antibody production *in vitro* in the presence of CD40L, including IgM, IgG1, IgG2 and IgA, with antibody titers modulated by IL-4 and IL-10 (28). DNA release caused by irradiation, combined with IL-15, also promotes proliferation and antibody production by IgM memory B cells and class-switch memory B cells (36). We are at the early stage of characterizing B cell function in S15-DKO mice and further investigation is underway to elicit antigen-specific humoral immune response. This is particularly important since the formation of anti-drug antibodies (ADA) has been observed in biotherapeutic treatments, leading to loss of drug efficacy and hypersensitivity reactions (37, 38). There has been no reliable way to predict the immunogenicity of monoclonal antibodies. With the high number of B cells, S15-DKO mice may serve as a promising next-generation *in vivo* model to test patient-specific ADAs and the efficacy of protein therapeutics.

Beyond lymphoid populations, we observed evidence of improved myeloid cell engraftment in S15-DKO mice, including DCs and macrophages. PBMC-engrafted DKO mice rarely exhibit detectable human DC populations in the spleen (prior unpublished results), but a higher proportion of S15-DKO mice contained measurable human CD3^-^CD19^-^HLA-DR^+^CD11C^+^ DCs in this study. Similarly, we detected significantly higher numbers of CD3^-^CD19^-^HLA-DR^+^CD64^+^ macrophages in S15-DKO spleens compared to DKO spleens. These findings are particularly important because myeloid cells serve critical functions in orchestrating adaptive immune responses, presenting tumor antigens, and responding to immunotherapies. SCF, GM-CSF and IL-3 are essential for myeloid cell differentiation and function (39), and their expression in S15-DKO mice likely creates a more permissive environment for human myeloid cell survival and expansion. Thus, tumor-bearing S15-DKO mice may be used for efficacy and safety testing of chimeric antigen receptor-engineered DCs (CAR-DCs) combined with CAR-T therapy (40).

Although irradiation was not required for PBMC engraftment in the S15-DKO model, it accelerated immune reconstitution and enhanced absolute immune cell counts. The most dramatic effects were observed for B cells and NK cells, which showed substantially higher engraftment levels in irradiated S15-DKO mice compared to non-irradiated controls. Irradiation likely creates additional space for engraftment by depleting residual mouse innate immune cells. Mouse cell death releases damage-associated molecular patterns, which are immunogenic, mediate sterile inflammation and promote human immune cell expansion (41–43), and our results are consistent with prior reports (44). The accelerated engraftment in irradiated mice offers practical advantages for preclinical studies by shortening experimental timelines. Furthermore, irradiation may enhance the function of engrafted immune cells with higher levels of IFN-γ and IL-10, as has been previously observed (44). Although subset-specific functional assays were out of scope for this study, the increased cytokine release in irradiated versus non-irradiated Raji cell-bearing PBMC-engrafted DKO mice is consistent with a functional benefit of irradiation for T cells and other immune subsets (44). At the same time, irradiation may exacerbate xenoreactivity and accelerate GvHD, as we observed in donor 8094 (but not in donor 0877), even though mouse MHC class I and II, the main driving force of acute GvHD, are absent in S15-DKO mice (19). Mature B cells, monocytes, and dendritic cells that can present antigen to T cells may explain the xenoreactivity once PBMCs are introduced into S15-DKO mice. Improved tumor clearance was observed in S15-DKO mice only after irradiation, likely due to higher PBMC engraftment in early timepoints compared to non-irradiated mice. Without irradiation, the lower engraftment level of PBMCs in S15-DKO reduces the overall magnitude of the alloantigen-specific T cell response to the Raji cells as compared to irradiated mice. In addition, S15-DKO mice may serve as a more favorable host for Raji cells growth compared to DKO mice, due to *IL15* expression in S15-DKO mice. Raji cells, a human B cell lymphoma cell line, express IL-15 receptor α (45, 46), which has high affinity to IL-15. IL-15 has previously been shown to promote proliferation of malignant B cell isolated from patients with B-cell chronic lymphocytic leukemia (47, 48). With IL-15-driven tumor growth and lower engraftment without irradiation, we did not observe improved tumor control in non-irradiated S15-DKO mice before PBMC engraftment.

The enhanced response of PBMC-humanized S15-DKO mice to anti-CD3xCD19 BiTE therapy compared to DKO mice supports its use as a sensitive immuno-oncology preclinical model. This increased sensitivity derives from the increased reconstitution of human immune cell diversity in S15-DKO mice, including T cells, B cells, NK cells, macrophages and dendritic cells. Raji cells also engraft better in S15-DKO compared to DKO. The concentration of anti-CD3xCD19 BiTE used in our experiments, 0.1 mg/kg, is very high based on our previous results (23). Therefore, even accounting for a bigger antigen sink from CD19^+^ Raji cells and normal B cells, sufficient BiTE should remain available to drive T cell engagement and cytotoxicity. However, there is a limitation in using a PBMC-engrafted model to test BiTE efficacy. The decreased tumor burden at later time points (SD 11 in our study) is not solely due to the BiTE-specific anti-tumor effect, since PBMC-mediated allogeneic response also clears tumor, as observed in the PBS groups. Therefore, setting up appropriate internal controls is extraordinarily important. Notably, CRS is a severe side effect of BiTE therapies that limits their clinical utility (22). The crucial driver of CRS is IL-6. IL-6, together with IL-8 and MIP-1A, which are commonly increased in clinical CRS, were significantly higher in Raji-bearing S15-DKO mice compared to Raji-bearing DKO mice after anti-CD3xCD19 BiTE treatment (49). Host macrophages are thought to play a central role in the pathophysiological mechanisms of CRS after T cell activation (49). The ability of S15-DKO mice to support DC and macrophage engraftment makes them a more physiologically relevant platform for evaluating CRS. The biological rationale for using S15-DKO mice to evaluate anti-tumor activity and cytokines release differs and overlaps. Anti-tumor activity of anti-CD3xCD19 BiTE is primarily improved by *IL15* expression in S15-DKO mice. IL-15 not only improves CD8^+^ memory T cells survival and proliferation, but also increases CD8^+^ T cells cytotoxic effector functions and cytokines production (50). IL-15 administration has been shown to increase anti-tumor activity both *in vitro* and *in vivo* (51–53). Activated CD4^+^ T cells may also enhance CD8^+^ T cells cytotoxicity by licensing dendritic cells through CD40L:CD40 interactions and cytokines production, such as IL-10 (54). In contrast, higher cytokines induction observed after anti-CD3xCD19 BiTE treatment is dependent on the human *KITLG*, *CSF2*, *IL3* as well as *IL15* expression in S15-DKO mice. The donor variability in cytokine response in these PBMC-humanized models could provide insight into strategies for enhancing BiTE efficacy while minimizing toxicity.

Although we have made progress in generating a better mouse strain for PBMC humanization with S15-DKO mice, the model does have limitations. First, more in-depth studies are needed to provide direct evidence for functionality of the engrafted human immune cells, including B cells, NK cells and myeloid cells. We focus on quantifying the immune cells engrafted in the mice and defining the diversity of immune cells. Further *in vitro* and *in vivo* studies are needed to assess their functionality. Second, to enable a broader evaluation of several human immune cell populations, the breadth of markers used to characterize cell identity and phenotype, particularly for myeloid cells, was more limited. Ongoing studies are expanding the evaluation of the innate immune cells and B cells by further characterizing their subtypes, maturation status and function. Third, S15-DKO mice mainly retain diverse immune cell populations during the early post-engraftment period. The number of B cells and NK cells in the blood dropped to low levels at 5 to 6 weeks post-PBMC engraftment, and T cells also started to decline in 5 to 6 weeks post-engraftment depending on the donor. PBMC engraftment in the spleen also decreased at the end of study with no difference between S15-DKO and DKO for most donors (data not shown). The S15-DKO advantage may therefore reflect accelerated kinetics rather than a sustained increase in reconstitution. This phenomenon correlates with the biology nature of PBMCs we engrafted. PBMC engraftment introduces mature immune cells without a self-renewing, hematopoietic progenitor pool. Human *IL3*, *CSF2*, *KITLG* and *IL15* expressed in S15-DKO mice enhance the survival and expansion of human immune cells including myeloid and NK lineages during the initial engraftment phase, but most mature T cells, B cells, NK and myeloid cells die off following xenogeneic activation-induced apoptosis. Only a minority of T cells become long-lived memory cells in the blood. It may therefore be advisable to use S15-DKO mice within 5 to 6 weeks post-PBMC engraftment to build disease models with diverse immune cell populations.

In conclusion, PBMC-engrafted S15-DKO mice represent an advancement in humanized mouse technology, combining robust immune cell engraftment with practical experimental timelines. The enhanced numbers of human NK cells, and of human B cell and myeloid cells for specific donors, coupled with sensitive responses to immunotherapy treatment, establish S15-DKO mice as a new preclinical platform for evaluating next-generation cancer immunotherapies.

## Data Availability

The original contributions presented in the study are included in the article/**Supplementary Material**. Further inquiries can be directed to the corresponding author.
