# Supplementary material for: Enhanced diversity of human immune cells in a PBMC-engrafted NSG-SGM3-IL15-DKO (S15-DKO) immunodeficient mouse model
Source: Front Immunol. 2026 Oct 27;17:1967450. doi: 10.3389/fimmu.2026.1967450 (PMC13748356; doi:10.3389/fimmu.2026.1967450)
Supplement: Supplementary file 1 [file DataSheet1.docx]

Supplementary Material

# 1 Supplementary Figures

**Supplementary Figure 1.** **Gating strategy and liver H&E analysis. (A)** An example of gating strategies used to identify live human CD45^+^ cells, CD19^+^ B cells, CD3^+^CD4^+^ T cells, CD3^+^CD8^+^ T cells, CD3^-^CD19^-^CD56^+^ natural killer cells, CD3^-^CD19^-^HLA-DR^+^CD64^+^ macrophages and CD3^-^CD19^-^HLA-DR^+^CD11c^+^ dendritic cells from a spleen. **(B)** Representative H&E staining of liver (20X, scale bar = 100 µm) from S15-DKO mouse and DKO mouse engrafted with donor 1309 on SD 28.

**Supplementary Figure 2. Representative flow cytograms and anti-human CD11c immunohistochemistry staining in the liver.** **(A)** human CD45^+^ cells, **(B)** CD19^+^B cells, **(C)** CD4^+^ and CD8^+^ T cells, **(D)** CD3^-^CD19^-^CD56^+^ natural killer (NK) cells in the peripheral blood from S15-DKO and DKO mice engrafted with donor 6184 on study day (SD) 28, and **(E)** CD3^-^CD19^-^HLA-DR^+^CD64^+^ macrophages and CD3^-^CD19^-^HLA-DR^+^CD11c^+^ dendritic cells (DCs) in the spleen from S15-DKO and DKO mice engrafted with donor 1309 on SD 28. **(F)** Representative flow cytograms of CD4^+^HLA-DR^+^ T cells and CD8^+^HLA-DR^+^ T cells from S15-DKO mice and DKO mice engrafted with donor 5822 on SD14. Percentage of CD4^+^HLA-DR^+^ T cells and CD8^+^HLA-DR^+^ T cells engrafted with **(G)** donor 1309, **(H)** donor 5822 and **(I)** donor 6184. **(J)** Representative anti-human CD11c immunohistochemistry staining of liver (40X, scale bar = 100 µm) from S15-DKO mice and DKO mice engrafted with donor 1309 on SD 28. Two-way ANOVA analysis (full model) with Sidak’s multiple comparison within each row was conducted in (G)-(I), * *p*<0.05, ** *p*< 0.01, *** *p*<0.001 and **** *p*<0.0001.

**Supplementary Figure 3.** **Body weight, survival and engraftment of peripheral blood mononuclear cells (PBMCs) in the spleens of S15-DKO and DKO mice. (A**) Body weight and **(B)** Survival of S15-DKO (red inverted triangles) and DKO (black circles) mice engrafted with PBMCs from donors (left to right) 0952, 2870, 3161, 2786 and 3643. **(C)** Human CD45^+^ cell counts, **(D)** CD19^+^ B cells and **(E)** CD19^+^CD138^+^ plasma cell counts in the spleens on SD 21. **(F)** Total human IgG in the plasma from S15-DKO and DKO mice engrafted with donor 1309 on SD 28. Data are presented as mean ± SEM, unpaired t test with Welch’s correction was performed, * *p*<0.05, ** *p*< 0.01, *** *p*<0.001 and **** *p*<0.0001.

**Supplementary Figure 4. PBMC engraftment in irradiated mice with lymphoma cell and breast cancer cell xenografts. (A)** Human CD45^+^ cell counts in blood and **(B)** human CD45^+^ cells, **(C)** CD3^+^ T cells, **(D)** CD19^+^ B cells, **(E)** CD56^+^ NK cells, **(F)** CD3^-^CD19^-^HLA-DR^+^CD11c^+^ DCs and **(G)** CD3^-^CD19^-^HLA-DR^+^CD64^+^ macrophages in the spleen on SD 9 in S15-DKO (red inverted triangles) and DKO (black circles) mice after cancer xenografts (left to right: PBS control, Raji lymphoma cells and MDA-MB-231 breast cancer cells) and PBMC engraftment. Tumor burden of (H) Raji lymphoma cells and (I) MDA-MB-231 cells on SD -1 to SD 8 in irradiated S15-DKO and DKO mice. Data are presented as mean ± SEM. Two-way ANOVA analysis (full model) with Sidak’s multiple comparison within each row was performed, * *p*<0.05, ** *p*< 0.01, *** *p*<0.001 and **** *p*<0.0001.

**Supplementary Figure 5. Anti-CD3xCD19 BiTE-induced cytokine release in irradiated or non-irradiated DKO mice bearing lymphoma xenografts.** Plasma levels of **(A)** interferon (IFN)-γ, **(B)** tumor necrosis factor (TNF), **(C)** interleukin (IL)-10, **(D)** IL-6, **(E)** IL-4 and **(F)** IL-2 are shown for Raji cell-bearing (2x10^6^ cells/mouse), PBMC-engrafted DKO mice (donor 1149, unfilled bars; donor 2134, filled bars) treated with PBS (unfilled circles) or anti-CD3xCD19 BiTE (filled circles). Data are shown as mean ± SEM. One-way ANOVA analysis with multiple comparisons, * *p*<0.05, *** *p*<0.001 and **** *p*<0.0001.

**Supplementary Figure 6. Anti-CD3xCD19 BiTE-induced cytokine release in S15-DKO and DKO mice with lymphoma cell xenografts.** Plasma levels of **(A)** sCD40L, tumor necrosis factor α (TNF α), granulocyte-macrophage colony-stimulating factor (GM-CSF), IL-2, IL-3, IL-4, IL-5, IL-9 and IL-17A, and **(B)** Macrophage-derived chemokine (MDC), monocyte chemoattractant protein-1(MCP-1), monokine induced by gamma interferon (MIG)/CXCL9, macrophage inflammatory protein-1α (MIP-1A), MIP-1B, and IL-8 in S15-DKO (red inverted triangles) or DKO (black circles) engrafted with PBMCs from donor 8130 (filled bars) or 9585 (unfilled bars) after exposure to PBS (unfilled symbols) or anti-CD3xCD19 BiTE (filled symbols). Data are shown as mean ± SEM. One-way ANOVA analysis with multiple comparisons, * *p*<0.05, ** *p*< 0.01, *** *p*<0.001 and **** *p*<0.0001.

**Supplementary Table 1** **PBMC donor information**

| **Donor ID** | **Age** | **Sex** | **Ethnicity** | **Vendor** | **Used in experiment** |
| --- | --- | --- | --- | --- | --- |
| 877 | 21 | Female | Caucasian | StemCell | Figure 4 and 5 (n=6 irradiated S15-DKO, n=13 non-irradiated S15-DKO and n=10 irradiated DKO) |
| 952 | 41 | Male | African American | Sanguine | Supplementary Figure 3 (n=20/strain) |
| 1149 | 43 | Male | Hispanic | Sanguine | Figure 4 (n=5 irradiated S15-DKO and n=4 non-irradiated S15-DKO) |
| 1309 | 54 | Female | Caucasian | BioIVT | Figure 1 and 2 (n=20/strain) |
| 1610 | 32 | Male | African American | AllCells | Figure 1 and 2 (n=20/strain) |
| 2134 | 36 | Male | African American | AllCells | Figure 4 (n=5 irradiated S15-DKO and n=5 non-irradiated S15-DKO) |
| 2786 | 30 | Male | Caucasian | Sanguine | Supplementary Figure 3 (n=20/strain) |
| 2870 | 29 | Male | Caucasian | AllCells | Supplementary Figure 3 (n=20/strain) |
| 3161 | 29 | Male | Pacific islander | AllCells | Supplementary Figure 3 (n=20/strain) |
| 3643 | 26 | Male | Other | AllCells | Supplementary Figure 3 (n=20/strain) |
| 5822 | 27 | Male | Caucasian | AllCells | Figure 1 and 2 (n=20/strain) |
| 6184 | 39 | Female | Caucasian | AllCells | Figure 1 and 2 (n=20/strain), Figure 3 (n=7 in PBS and n=9 in Raji group on SD10 and n=4 in PBS and n=5 in Raji group on SD21) |
| 8094 | 25 | Male | Caucasian | Lonza | Figure 4 and 5 (n=13 irradiated S15-DKO, n=12 non-irradiated S15-DKO and n=13 irradiated DKO) |
| 8130 | 21 | Male | Other | Lonza | Figure 6 (n=7 in PBS of S15-DKO, n=6 in BiTE of S15-DKO, n=6 in both PBS and BiTE of DKO) |
| 9585 | 42 | Male | African American | Lonza | Figure 6 (n=6 in PBS and BiTE of S15-DKO, n=6 in PBS of DKO and n=7 in BiTE of DKO) |

**Supplementary Table 2** **B cell engraftment in S15-DKO and DKO mice**

|  | **Donor ID** | **Irradiation** | **CD19^+^ engraftment** | **CD19^+^ CD138^+^ engraftment** |
| --- | --- | --- | --- | --- |
| 1 | 1309 | N | S15-DKO > DKO | N/A |
| 2 | 5822 | N | No significant difference | N/A |
| 3 | 6184 | N | S15-DKO > DKO | N/A |
| 4 | 0877 | Y | No significant difference | N/A |
| 5 | 8094 | Y | S15-DKO > DKO | N/A |
| 6 | 0952 | N | No significant difference | S15-DKO > DKO |
| 7 | 2870 | N | S15-DKO > DKO | S15-DKO > DKO |
| 8 | 3161 | N | S15-DKO > DKO | S15-DKO > DKO |
| 9 | 2786 | N | S15-DKO > DKO | S15-DKO > DKO |
| 10 | 3643 | N | No significant difference | S15-DKO > DKO |
